# Phenotypic Clustering in Non-Cystic Fibrosis Bronchiectasis Patients: The Role of Eosinophils in Disease Severity

**DOI:** 10.3390/ijerph18168431

**Published:** 2021-08-10

**Authors:** Xuejie Wang, Carmen Villa, Yadira Dobarganes, Casilda Olveira, Rosa Girón, Marta García-Clemente, Luis Máiz, Oriol Sibila, Rafael Golpe, Rosario Menéndez, Juan Rodríguez-López, Concepción Prados, Miguel Angel Martinez-García, Juan Luis Rodriguez, David de la Rosa, Xavier Duran, Jordi Garcia-Ojalvo, Esther Barreiro

**Affiliations:** 1 Lung Cancer and Muscle Research Group, Pulmonology Department, Hospital del Mar-IMIM, Parc de Salut Mar, 08003 Barcelona, Spain; xue62392@gmail.com; 2 Department of Medicine, Universitat Autònoma de Barcelona (UAB), 08193 Barcelona, Spain; 3 Respiratory Department, Clínica Fuensanta, 28027 Madrid, Spain; mcvillac@gmail.com (C.V.); yadira.dobarganes@yahoo.es (Y.D.); 4 Respiratory Department, Instituto de Investigación Biomédica de Málaga (IBIMA), Hospital Regional Universitario de Málaga, Universidad de Málaga, 29010 Málaga, Spain; casi1547@separ.es; 5 Respiratory Department, Instituto de Investigación Sanitaria, Hospital Universitario de la Princesa, 28006 Madrid, Spain; rmgiron@gmail.com; 6 Respiratory Department, Hospital Universitario Central de Asturias, 33011 Oviedo, Spain; mgclemen@gmail.com; 7 Respiratory Department, Hospital Ramon y Cajal, 28034 Madrid, Spain; luis.maiz@salud.madrid.org; 8 Respiratory Department, Hospital Clínic, 08036 Barcelona, Spain; OSIBILA@clinic.cat; 9 Centro de Investigación en Red de Enfermedades Respiratorias (CIBERES), Instituto de Salud Carlos III (ISCIII), 28029 Madrid, Spain; mianmartinezgarcia@gmail.com; 10 Respiratory Department, Hospital Lucus Augusti, 27003 Lugo, Spain; rafa898@separ.es; 11 Respiratory Department, Hospital Universitario y Politécnico La Fe, 46026 Valencia, Spain; rosmenend@gmail.com; 12 Respiratory Department, Hospital San Agustin, Avilés, 33401 Asturias, Spain; juan_rodriguezl@hotmail.com; 13 Respiratory Department, Hospital la Paz, 28046 Madrid, Spain; conchaprados@gmail.com; 14 Respiratory Department, Instituto de Investigación Sanitaria del Hospital Clínico San Carlos (IdISSC), Hospital Clínico San Carlos, 28040 Madrid, Spain; jlrhermosa@yahoo.es; 15 Departament of Medicine, Universidad Complutense de Madrid, 28040 Madrid, Spain; 16 Respiratory Department, Hospital Santa Creu I Sant Pau, 08041 Barcelona, Spain; david.rosa23@gmail.com; 17 Scientific and Technical Department, Hospital del Mar-IMIM, 08003 Barcelona, Spain; xduran@imim.es; 18 Department of Health and Experimental Sciences (CEXS), Universitat Pompeu Fabra (UPF), 08002 Barcelona, Spain; jordi.g.ojalvo@upf.edu

**Keywords:** non-cystic fibrosis bronchiectasis, eosinophil counts, biostatistical analyses, multivariate analyses, clinical outcomes, phenotypic clusters, disease severity scores

## Abstract

Whether high blood eosinophil counts may define a better phenotype in bronchiectasis patients, as shown in chronic obstructive pulmonary disease (COPD), remains to be investigated. Differential phenotypic characteristics according to eosinophil counts were assessed using a biostatistical approach in a large cohort study from the Spanish Online Bronchiectasis Registry (RIBRON). The 906 patients who met the inclusion criteria were clustered into two groups on the basis of their eosinophil levels. The potential differences according to the bronchiectasis severity index (BSI) score between two groups (Mann–Whitney *U* test and eosinophil count threshold: 100 cells/µL) showed the most balanced cluster sizes: above-threshold and below-threshold groups. Patients above the threshold exhibited significantly better clinical outcomes, lung function, and nutritional status, while showing lower systemic inflammation levels. The proportion of patients with mild disease was higher in the above-threshold group, while the below-threshold patients were more severe. Two distinct clinical phenotypes of stable patients with non-cystic fibrosis (CF) bronchiectasis of a wide range of disease severity were established on the basis of blood eosinophil counts using a biostatistical approach. Patients classified within the above-threshold cluster were those exhibiting a mild disease, significantly better clinical outcomes, lung function, and nutritional status while showing lower systemic inflammatory levels. These results will contribute to better characterizing bronchiectasis patients into phenotypic profiles with their clinical implications.

## 1. Introduction

Non-cystic fibrosis (CF) bronchiectasis is a highly prevalent chronic respiratory disease in which the airways are permanently dilated as a result of many different etiologies [1,2,3]. The function of the immune response against infections is of paramount importance in the development of bronchiectasis [4]. Thus, patients with bronchiectasis present acute exacerbations frequently [5,6,7,8,9]. The early characterization of bronchiectasis patients who are at risk of experiencing exacerbations and/or other clinical symptoms, including systemic alterations, is needed in clinical settings. Recently, high-resolution computerized tomography scans have been commonly used in clinics in order to monitor disease progression [7,10]. On the other hand, the sputum analysis and blood tests, in which the nutritional parameters such as serum albumin [11] and a great variety of inflammatory biomarkers are detected, are also used routinely in the clinics. 

As the abovementioned immune deficiency and host defense are major contributors to the development of bronchiectasis in patients, neutrophils are counted among the most relevant players in the fight against infection in bronchiectasis patients [12]. The impaired function of the immune system as identified by a reduction in the neutrophil oxidative burst and/or the phagocytic function has been proposed as relevant contributor to the pathogenesis of bronchiectasis in certain patients [13]. Primary antibody deficiencies, such as common variable immunodeficiency or IgG subclass deficiency, were also associated with the development of bronchiectasis [14]. A decrease in the number of lymphocytes may also participate in the pathogenesis of bronchiectasis, as these cells play a major role in the host adaptive immune response [15]. Other inflammatory cell types such as eosinophils may also play a role in the prognosis and in the response to treatment in these patients [16,17,18,19]. For instance, non-CF bronchiectasis patients with high levels of blood eosinophils were shown to respond better to treatments with different biological agents [20,21] and to inhaled corticosteroids [17,22]. Similarly, patients with COPD exhibiting blood eosinophil counts lower than 2% were also those showing an inadequate response to inhaled corticosteroids [23]. On the other hand, high eosinophil counts were associated with better outcomes and survival in COPD patients of the CHAIN (COPD History Assessment In SpaIN) and BODE (body mass index (B), degree of airflow obstruction (O), functional dyspnea (D), and exercise capacity (E)) cohorts [24]. Whether high blood eosinophil levels may also help identify a less severe phenotype in patients with bronchiectasis remains to be elucidated. 

Other diagnostic tools such as lung function and exercise testing also offer a great deal of information on the status and performance of non-CF bronchiectasis patients [25,26]. Whether the analysis of the datasets obtained from the different tests may assist clinicians in better characterizing their patients and establishing potential phenotypes remains an open question. Biostatistical approaches, which combine the use of software tools with complex biological datasets, may be very helpful in identifying different phenotypes in patients with non-CF bronchiectasis. On the other hand, the management of patients with bronchiectasis mainly focuses on the control of the symptoms with the aim to prevent acute exacerbations and delay disease progression. The phenotypic stratification of patients with bronchiectasis into those with a greater risk of experiencing acute exacerbations and hospitalizations is of paramount importance [27]. Moreover, severity indices represent useful tools that help predict the disease prognosis and all-cause mortality [28]. In this regard, a recent meta-analysis demonstrated that a cut-off value greater than 5 reliably predicted hospitalizations and all-cause mortality, according to the FACED and bronchiectasis severity index (BSI) scores [29].

On the basis of the current knowledge, the present investigation sought to tease out differential phenotypic characteristics on the basis of blood cells, particularly eosinophil counts, in a large cohort study based on the Spanish Online Bronchiectasis Registry (RIBRON). Additionally, the eosinophil clusters of the patients were further stratified into three disease severity groups following well-validated indices. Thus, our objectives were: (1) to identify a cut-off value of the blood eosinophil counts among the patients included in this registry that could discriminate differential phenotypic clusters; (2) to analyze the potential differences in the several clinical and analytical parameters between the clusters; (3) to stratify the clusters according to the disease severity following the EFACED, BSI, and FACED indices; and (4) to explore the potential associations between eosinophil and other blood cells with the disease severity scores and the number of hospitalizations and exacerbations. 

## 2. Methods

### 2.1. Study Design

This was a multicenter, prospective, and observational study, in which 43 centers from Spain participated within the frame of the RIBRON database between February 2015 and October 2019 [25,30,31]. Strengthening the Reporting of Observational studies in Epidemiology (STROBE) reporting guidelines was used to design the current investigation [32]. The quality of the data introduced in the registry was always monitored and ensured by an external contract research organization (CRO). 

### 2.2. Study Population

**Figure 1:**
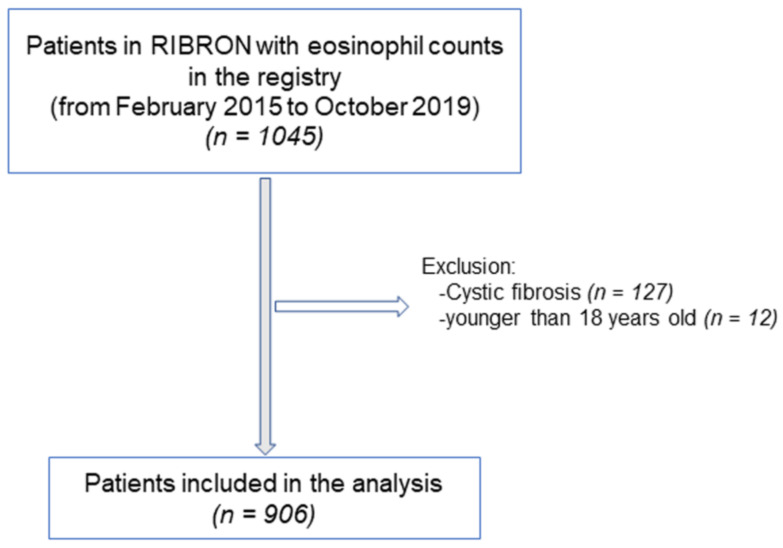
Flowchart of the study patients.

The flowchart of the patient recruitment for the purpose of the study is depicted in Figure 1. The inclusion criteria were adult patients who had been diagnosed with non-CF bronchiectasis by means of high-resolution computerized tomography (HRCT) [7,10,30,31,33,34]. In the current investigation, 906 patients were analyzed from the registry. General clinical data such as anthropometry, smoking history, lung function, hemogram, inflammatory blood cells, and nutritional parameters were analyzed using custom data analysis software tools. All the included patients were stable and had not reported any acute exacerbations at least in the last four weeks prior to entry in the study. The exclusion criteria included traction bronchiectasis and/or cystic fibrosis and ages younger than 18 years old. The research followed the guidelines of the World Medical Association for Research in Humans (seventh revision of the Declaration of Helsinki, Fortaleza, Brazil, 2013) [35]. Ethical approval was obtained from the Ethics Committee at the Hospital Josep Trueta Girona (# 001-2012, Hospital Universitari Dr. Josep Trueta, Girona, Spain) in the coordinating center and in the local participating centers. All the patients signed the informed written consent to participate in the registry. The information remained confidential at all times, and no personal information related to any of the participants was introduced into the registry. 

### 2.3. Study Variables and Scores

The following clinical variables and parameters were obtained from all the study patients: etiology of the non-CF bronchiectasis; anthropometry (age, sex, and body mass index); lung function; chronic colonization by *Pseudomonas aeruginosa*; chronic colonization with other microorganisms; radiologic extension; dyspnea; the number of exacerbations and hospitalizations for exacerbations in the previous year; the Charlson index; smoking history; nutritional status; and systemic inflammatory cells and markers. Additionally, three multidimensional scales of the severity of bronchiectasis were calculated on the basis of those variables: FACED [36], EFACED [37], and bronchiectasis severity index (BSI) [38].

### 2.4. Severity Scores

The EFACED score (number of exacerbation with hospital admission in the previous year (E; cutoff, at least one number, 0 or 2 points), FEV_1_ percent predicted (F; cutoff, 50%; 0 or 2 points), age (A; cutoff, 70 years; 0 or 2 points), chronic colonization by *Pseudomonas aeruginosa* (C; yes, 0 or 1 points), radiological extension (E; number of lobes affected; cutoff, two lobes; 0 or 1 points), and dyspnea (D; cutoff, grade 2 on the Medical Research Council dyspnea scale; 0 or 1 points)) divided bronchiectasis into three groups according to the severity scores (mild, 0–3 points; moderate, 4–6 points; and severe, 7–9 points).

The FACED score, which was calculated based on the FEV_1_ percent predicted, age, chronic colonization by *Pseudomonas aeruginosa*, radiological extension, and dyspnea, divided bronchiectasis into three groups (mild, 0–2 points; moderate, 3 to 4 points; and severe, 5–7 points).

The BSI score (age (maximum value: 6 points), BMI (maximum value: 2 points), FEV_1_ (maximum value: 3 points), hospital admission prior to study (maximum value: 5 points), exacerbations prior to the study (maximum value: 2 points), MRC dyspnea scale (maximum value: 3 points), chronic colonization by *Pseudomonas aeruginosa* (maximum value: 3 points), chronic colonization by other microorganisms (maximum value: 1 point), and radiological extension (maximum value: 1 point)) was evaluated to divide bronchiectasis into three groups according to the severity scores (mild, 0–4 points; moderate, 5–8 points; and severe, ≥9 points).

### 2.5. Patient Clustering

The 906 patients who met the inclusion criteria were divided into two groups, depending on whether their eosinophil levels were lower or higher than a given threshold. To determine the optimal eosinophil threshold in a number of cells, we compared the distributions of the BSI scores for the two groups of patients and selected a threshold for which BSI score difference between the two groups was statistically significant while maintaining a certain balance between the sizes of the two groups (see the corresponding results below). The similarity between the BSI distributions was assessed by means of the Mann–Whitney *U* test. We selected the highest threshold of eosinophils (100 cells/µL) that evidenced a statistically significant difference in the BSI scores between the above- and the below-threshold patients. The range of the eosinophil counts was within the normal limits (20–500 cells/µL) for most of the patients, except for 72 patients whose levels were lower than 20 cells/µL and 50 patients with levels greater than 500 cells/µL. 

### 2.6. Statistical Analysis

The normality of the distribution of the study variables was assessed using the Kolmogorov–Smirnov test. Comparisons between the study groups were made for all the study variables. The differences between the two clusters of patients (above-threshold and below-threshold) were assessed using the Student’s *t*-test for the quantitative variables and the chi-square test for the categorical variables. The study variables are presented as the mean (standard deviation) in the tables. Histograms were used to represent the distribution of the dichotomized eosinophils (above versus below threshold) for each severity variable (EFACED, BSI, and FACED). The correlations between the clinical and biological variables were explored using the Pearson’s correlation coefficient. Correlations between the clinical and biological variables were explored using the Pearson’s correlation coefficient: all the patients together as a whole and each cluster of patients were analyzed separately. 

A multivariate logistic regression, taking as an outcome the variable defined by BSI ≥ 9 versus BSI < 9, was used to evaluate the associations of the dichotomized eosinophils. Additionally, the clinically meaningful confounders were selected by the investigators and were the following: chronic colonization by *Pseudomonas aeruginosa*, the Charlson index, the erythrocyte sedimentation rate (ESR), the total number of leukocytes and neutrophils, fibrinogen, hemoglobin, hematocrit, the total protein and albumin levels, and C-reactive protein (CRP). Statistical analyses were performed using Stata 15.1 (StataCorp LLC, College Station, TX, USA). For all the analyses, the statistical significance was established at *p* < 0.05. 

## 3. Results

### 3.1. Cut-off Analysis

**Figure 2:**
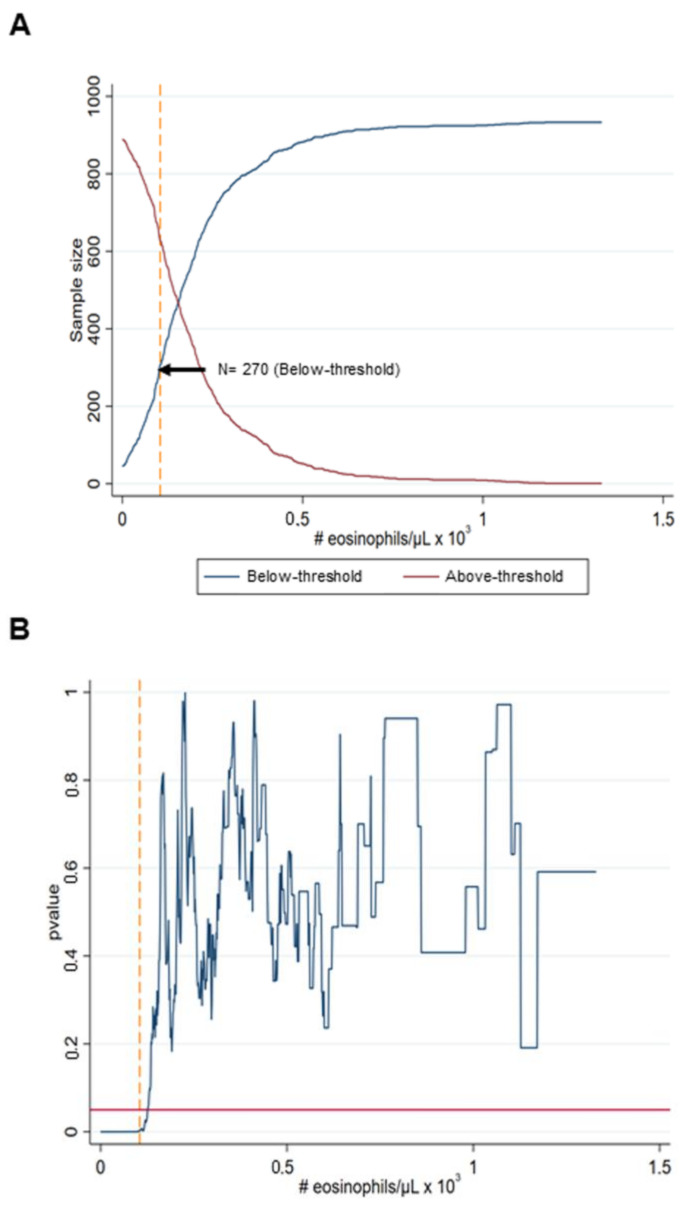
Selection of the eosinophil threshold for patient clustering. (**A**) Number of patients in the below-threshold cluster for increasing the eosinophil thresholds. The point indicated by the black arrow corresponds to the percentage of the patients’ threshold in the below-threshold, which led to the most balanced cluster sizes. (**B**) Statistical significance resulting from the Mann–Whitley *U* test as a measure by the corresponding *p*-value for varying thresholds (in terms of the percentage of patients belonging to the below-threshold cluster). The horizontal solid red line shows the statistical significance threshold *p* = 0.05, while the vertical yellow dashed line shows the optimal threshold of the eosinophil counts corresponding to the *p*-value.

A threshold for which 30% of the patients (270 out of 906) were in the below-threshold cluster led to the most balanced cluster sizes while maintaining a statistically significant separation between their degree of BSI scores (Figure 2A). The eosinophil count for that threshold was 100 cells/µL, which corresponded to a *p* < 0.01 (Figure 2B).

### 3.2. Anthropometry

The total body weight and body mass index (BMI) were significantly greater in the above-threshold than in the below-threshold patients (Table 1A). Ages did not differ between the two patient clusters. Similar results were encountered when COPD patients (*n* = 103) were excluded from the analysis (Table 1B). The BMI and body weight did not significantly correlate with any of the other study variables either clinical or analytical (data not shown). 

### 3.3. Disease Severity

In the above-threshold cluster of non-CF bronchiectasis patients compared to the below-threshold patients, the EFACED and BSI scores and the number of exacerbations and hospitalizations index were significantly lower but not the Charlson index (*p* = 0.1) (Table 1A). Similar results were observed when COPD patients were excluded from the analysis (Table 1B). 

**Figure 3:**
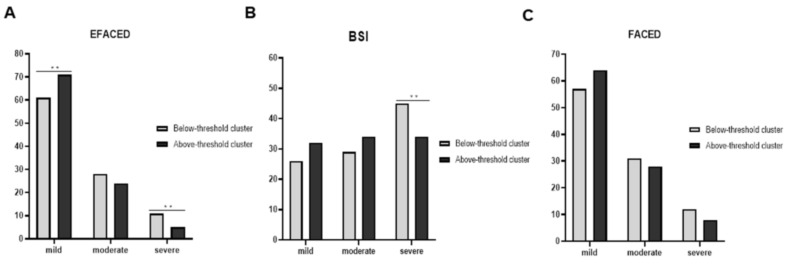
Histograms of the proportions of patients who were classified as mild–moderate–severe according to EFACED (**A**), BSI (**B**), and FACED (**C**) between the two clusters. EFACED: mild: 0–3, moderate: 4–6, and severe: 7–9; BSI: mild: 0–4, moderate: 5–8, and severe: ≥ 9; and FACED: mild: 0–2, moderate: 3 to 4, and severe: 5–7. Statistical significance: **, *p* < 0.01 between the patient groups.

**Figure 4:**
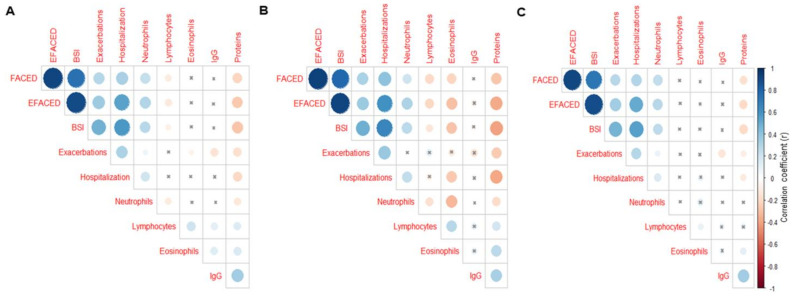
Correlation matrix of the disease severity and analytical variables, in which the positive correlations are represented in blue, while the negative correlations are represented in red: (**A**) all the study patients, (**B**) patients in the below-threshold cluster, and (**C**) patients in the above-threshold cluster. The intersection within the circle represents a *p*-value > 0.05. The color intensity and the size of the circle are proportional to the correlation coefficients, as indicated in the Y-axis on the right-hand side of the graph.

Compared to the below-threshold patients, in the above-threshold group, greater proportions of patients were classified as mild according to the EFACED score (Figure 3A). However, significantly smaller proportions of the above-threshold patients were classified as severe according to the EFACED and BSI scores (Figure 3A,B). According to FACED, no statistically significant differences were seen between the above- and below-threshold patients with either mild or moderate disease severity (Figure 3C). When all the patients were analyzed together, significant positive correlations were detected between the number of neutrophils and the variables hospitalizations, FACED, EFACED, and BSI, whereas a negative weak correlation was found between the exacerbations and eosinophil numbers (Figure 4A). Among the below-threshold cluster of patients, significant correlations were observed between the number of neutrophils and FACED, EFACED, BSI, and hospitalizations (Figure 4B). In the same cluster, however, negative associations were detected between the eosinophil numbers and FACED, EFACED, BSI, hospitalizations and almost significantly with the exacerbations (*p* = 0.076, Figure 4B). Additionally, in the below-threshold cluster, significant negative associations were also detected between the FACED, EFACED, BSI, exacerbations, hospitalizations, and systemic protein levels (Figure 4B). Among the above-threshold cluster of patients, significant positive correlations were detected between either FACED, EFACED, or BSI and the number of neutrophils (Figure 4C). Additionally, in the same cluster of patients, weak negative correlations were also detected between the systemic levels of the proteins and any of three severity scores (Figure 4C). 

### 3.4. Lung Function

The smoking history was similar between the two clusters (Table 1A). Furthermore, the airway obstruction, diffusion capacity, and airway trapping (*p* = 0.061) were significantly better in the above-threshold cluster than in the below-threshold group (Table 1A). Similar results were detected when patients with concomitant COPD were excluded from the analysis (Table 1B). 

### 3.5. Blood Inflammatory Markers

The levels of the blood cell neutrophils were significantly lower in the above-threshold cluster than in the below-threshold group, while the lymphocyte numbers were significantly higher (Table 2A). Nonetheless, the total number of eosinophils was significantly greater in the former than in the latter cluster of patients (Table 2A). When all the patients were analyzed together, the IgG levels positively correlated with the total protein levels (Figure 4A). Among the below-threshold cluster of patients, a negative association was observed between the number of eosinophils and those of neutrophils (Figure 4B). In the above-threshold cluster of patients, however, a significant positive correlation was observed between the number of eosinophils and that of lymphocytes (Figure 4C). A significant positive correlation was detected between the systemic protein levels and IgG in the above-threshold cluster of patients (Figure 4C). Moreover, the blood levels of IgG were also significantly higher in the above-threshold cluster than in the below-threshold group of patients (Table 2A). The protein levels of alpha-1 antitrypsin were normal for all the patients and did not significantly differ between the two clusters (Table 2A). The levels of the inflammatory parameters CRP, GSV, and fibrinogen did not significantly differ between the above-threshold and the below-threshold groups of patients (Table 2A). Furthermore, the hemogram parameters such as hemoglobin and hematocrit, along with the total protein and albumin levels, were significantly greater in the above-threshold cluster than in the below-threshold group of bronchiectasis patients. Similar results were observed when the patients with concomitant COPD were excluded from the analysis (Table 2B). 

### 3.6. Etiology of Non-CF Bronchiectasis

The percentage of patients that fell into each category of the etiologic condition of non-CF bronchiectasis was similar between the two clusters of patients (Table 3). The presence of COPD was similar in the study groups of patients (Table 3). 

### 3.7. Multivariate Analyses

**Figure 5:**
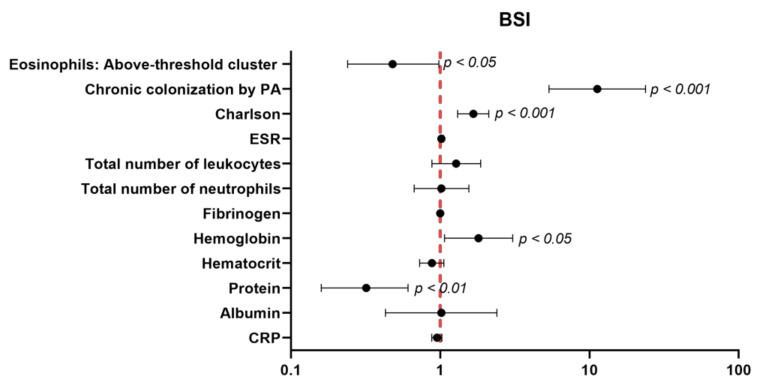
The independent association of dichotomized eosinophils with severe BSI. A multivariate regression analysis was used to perform the analysis. The confidence intervals and statistical significance are represented in the figure panel. The odds ratio is represented as black dots. The model was adjusted by chronic colonization by the PA, Charlson Index, ESR, total number of leukocytes, total number of neutrophils, fibrinogen, hemoglobin levels, hematocrit levels, protein, albumin, and CRP levels.

A statistically significant association of dichotomized eosinophils with severe BSI (odds ratio (OR) = 0.48; CI95%: 0.24–0.98, *p* value = 0.044) was found. Patients of the above-threshold group were those exhibiting a lower risk to attain greater BSI scores (OR < 1), as demonstrated by the multivariate model after adjusting for potential confounders (Figure 5). 

## 4. Discussion

The results obtained in the present study put the line forward on the existence of two different phenotypes of patients with stable non-CF bronchiectasis of a wide range of disease severities. The biostatistical approach used in the current investigation divided the population into two major clusters on the basis of the number of eosinophil counts above and below the established threshold of 30% (equivalent to 100 cells/µL) according to the threshold that best divided the study population into two groups. Thus, two major phenotypic clusters of stable patients with non-CF bronchiectasis of a wide range of disease severities were defined. The biostatistical analyses revealed the presence of the two different clinical phenotypes that are discussed below.

Importantly, from a clinical standpoint, patients with the above-threshold profile were those with statistically significant lower levels of EFACED and BSI scores, along with a lower number of exacerbations and hospitalizations for exacerbations. Furthermore, the below-threshold cluster of patients were those bearing a significantly more severe disease according to the EFACED and BSI scores, while the patients with a mild form of the disease were predominantly those stratified in the above-threshold cluster. The number of hospital admissions and the degree of airway obstruction were probably the most significant contributors to the differences seen between the EFACED and BSI stratifications of the two patient clusters. The two scores showed that the more severe patients were those classified in the below-threshold category. In the present study, the multivariate regression analyses evidenced that the patients of the above-threshold group were those exhibiting a lower risk to attain greater BSI scores.

Importantly, the lung function parameters, including spirometry and the diffusion capacity, were also significantly better among the patients classified within the above-threshold cluster than in the below-threshold patients. These are relevant results that imply that eosinophils may have a protective role in patients with non-CF bronchiectasis. In keeping with this, it has been demonstrated that eosinophils exert antibacterial properties in tissues [39]. Furthermore, low eosinophil counts (<100 cells/µL) increase the risk of pneumonia in COPD patients with bronchial colonization, particularly in those receiving treatment with inhaled corticosteroids [40]. In the present study, the below-threshold patients were not on systemic corticosteroid treatment, and the prevalence of concomitant severe diseases such as solid and hematologic cancers was significantly lower than that seen in patients the above-threshold level of the eosinophil counts. Hence, in the current investigation, it is possible to conclude that a relatively great number of eosinophils defined a very specific clinical phenotype characterized by better lung function parameters and lower disease severity, as quantified by the EFACED and BSI scores, together with a significantly lower number of acute events and hospital admissions during the previous 12 months prior to study entry. As far as we are concerned, these are novel findings in the characterization of the potential phenotypes among patients with stable non-CF bronchiectasis. 

As to the etiology of the bronchiectasis in the two study groups, no significant differences were detected between the above- and the below-threshold clusters. Specifically, the proportions of patients who also had concomitant COPD were similar in the two groups of bronchiectasis patients. It should be emphasized that those proportions were low (11%) compared to the total amount of the analyzed patients. Additionally, when COPD patients were excluded from the analysis of comparisons between the two patient clusters, similar results were seen for all the study variables. Interestingly, the smoking history was alike in the two clusters of patients, probably due to the low prevalence of COPD among the study population. 

The levels of the general inflammatory parameters, such as the number of inflammatory cells (total neutrophil levels), were significantly reduced in the above-threshold cluster of the stable non-CF bronchiectasis patients compared to the below-threshold group. In keeping with this, it has been previously reported that high levels of the cell and molecular inflammatory parameters were associated with poorer clinical outcomes, including a lung function assessment among the participants in a population-based study [41]. Importantly, the numbers and proportions of neutrophils were significantly reduced in the above-threshold compared to the below-threshold patients. Indeed, significant positive correlations were also found between any of the three severity scores and the total number of neutrophils in the above-threshold cluster. Importantly, in the below-threshold cluster of patients, the eosinophil numbers were inversely associated with all three disease severity scores, as well as with the hospitalizations, exacerbations, and neutrophil counts. Additionally, in the same cluster of patients, the protein levels were also negatively associated with all three disease scores, exacerbations, and hospitalizations. These results clearly demonstrated that bronchiectasis patients exhibiting low numbers of eosinophils were those showing greater values of disease severity scores and that low levels of systemic proteins were also associated with significantly worse outcomes. Hence, it would be possible to conclude that low eosinophil counts may be used as a biomarker of disease severity and exacerbation. These are novel relevant findings that warrant further attention in future studies aimed to tease out whether low eosinophil levels can predict the morbidity and mortality in bronchiectasis patients. 

On the other hand, it should be mentioned that COPD did not have any impact on the numbers of the inflammatory cell types, as shown in the analysis in which these patients were excluded. These findings suggest that bronchiectasis patients follow a rather neutrophilic pattern and that those with lower levels of eosinophils expressed greater numbers of neutrophils. These results may have implications in the resolution of airway and lung infections and in the disease progression in bronchiectasis patients, as also formerly demonstrated [42]. The results obtained in this study are also consistent with those reported in previous investigations in which large cohorts of COPD patients were also recruited [23,24]. In those studies [23,24], high levels of eosinophils were, indeed, associated with both better clinical outcomes, including survival and a greater response to inhaled corticosteroid treatment in COPD patients. Furthermore, a better response to several therapies, including biological agents [20,21] and inhaled corticosteroids [17,22], was observed in non-CF bronchiectasis patients with high levels of blood eosinophils. Collectively, the results reported so far in COPD [23,24] and bronchiectasis patients [17,20,21,22] and those obtained in the present study suggest that a high eosinophil profile may be protective in patients with chronic airway diseases. 

The nutritional parameters such as hematocrit and hemoglobin, along with the total protein and albumin levels, although within the normal range in both clusters of patients, were also significantly greater among the above-threshold cluster than in the below-threshold group of patients. Besides, a significant positive correlation was also detected between the total levels of the proteins and those of IgG among the above-threshold cluster of the patients and when all the patients were analyzed together. Furthermore, in the below-threshold cluster, negative associations were detected between the systemic protein levels and all three disease severity scores, hospitalizations, exacerbations, and neutrophil numbers. Collectively, these findings suggest that the nutritional status as measured by the total serum protein levels correlates well with the disease severity, as well as with the IgG levels, as previously demonstrated [43]. The total body weight or BMI, however, did not significantly correlate with any of the study variables. Although the underlying pathophysiology that may account for these findings remains obscure, it is possible to conclude that higher levels of blood eosinophils were associated with a better nutritional status and lung function, as also recently shown in Japanese patients with severe emphysema [44]. Further investigations will have to confirm these findings. 

### Study Critique

One of the strengths of this study is that there is a large number of well-characterized patients from 43 different centers across Spain whose registry was always strictly monitored by the CRO. Nonetheless, several limitations should also be acknowledged. The descriptive nature of the study may pose some difficulties at the time of analyzing the potential biological mechanisms accounting for the findings reported herein. Future investigations should focus on the elucidation of the underlying biology, whereby a relatively high level of eosinophils may exert protective effects in terms of the disease progression and prognosis in patients with non-CF bronchiectasis. Similar approaches may be applied in large population cohorts in which voluntary participants are being prospectively recruited, such as the cross-sectional UK Biobank [45,46]. The UK Biobank collects extensive phenotypic and genotypic features of the study participants, thus allowing researchers to address questions that require the use of large-scale databases of patients with chronic respiratory diseases [45,46]. The RIBRON, however, focuses more on the actual clinical and analytical data from the non-CF bronchiectasis patients. The stratification of the patients included in the RIBRON on the basis of the other clinical and/or analytical variables should also be assessed in future investigations. Finally, an external validation of our results would be also desirable in future investigations. 

## 5. Conclusions

Two distinct clinical phenotypes of stable patients with non-CF bronchiectasis of a wide range of disease severities were established on the basis of the blood eosinophil counts using a biostatistical approach. The patients classified within the above-threshold cluster were those exhibiting a mild disease, significantly better clinical outcomes, lung function parameters, and nutritional status while showing lower levels of the systemic inflammatory parameters. The low level of eosinophils revealed a specific phenotype that was associated with poorer clinical outcomes, such as disease severity and hospitalizations. Other inflammatory cell types or clinical parameters did not influence the disease severity in this cohort of patients. 

These are novel observations that warrant further attention to gain insight into the underlying pathophysiology accounting for the implications of greater blood eosinophil counts into the clinics in stable non-CF bronchiectasis. These results may contribute to better characterize bronchiectasis patients into phenotypic profiles tailored to customize personalized therapeutic strategies, particularly in those with significantly lower levels of eosinophils. 

## Figures and Tables

**Table 1:** (**A**) General characteristics of all the study patients. (**B**) General characteristics in the two clusters of bronchiectasis patients, excluding those with COPD.

**(A)**		
	**Below-Threshold Cluster**	**Above-Threshold Cluster**
	**(*N* = 270)**	**(*N* = 636)**
**Anthropometric variables**, $\bar{x}$**(SD)**		
Age, years	68.8 (13.8)	67 (15.1)
Body weight, kg	65.3 (15.1)	68.7 (14.4) **
BMI, kg/m^2^	25.2 (5.2)	26.4 (5) **
**Disease severity**, $\bar{x}$**(SD)**		
FACED score	2.21 (1.82)	2.07 (1.64)
EFACED score	3.11 (2.40)	2.65 (2.09) **
BSI	8.60 (4.96)	7.29 (4.51) ***
Exacerbations	1.82 (1.97)	1.39 (1.45) **
Hospitalizations	0.84 (1.30)	0.56 (1.38) **
Charlson Index	2.0 (1.71)	1.83 (1.53), *p* = 0.1
Chronic colonization by PA, N (%)	62 (23)	166 (26)
**Smoking history**		
Never smokers, N (%)	152 (56)	375 (59)
Current smokers, N (%)	28 (11)	58 (9)
Ex-smokers, N (%)	90 (33)	203 (32)
Packs-year, $\bar{x}$ (SD)	14.5 (23.9)	11.9 (21.1)
**Lung function**, $\bar{x}$**(SD)**		
FEV_1_, % predicted	71 (27)	75 (24) *
FVC, % predicted	83 (24)	86 (21), *p* = 0.065
FEV_1_/FVC, %	67 (13)	69 (13) **
DL_CO_, % predicted	76 (23)	85 (24) **
K_CO_, % predicted	77 (34)	77 (39)
RV, % predicted	137 (40)	138 (53)
TLC, % predicted	99 (20)	103 (20)
RV/TLC, %	52 (10)	49 (12), *p* = 0.061
(**B**)		
	**Below-Threshold Cluster**	**Above-Threshold Cluster**
	**(*N* = 236)**	**(*N* = 567)**
**Anthropometric variables,** $\bar{x}$ **(SD)**		
Age, years	67.8 (14.0)	66.0 (15.3)
Body weight, kg	64.4 (14.6)	68.1 (14.2) **
BMI, kg/m^2^	25.0 (5.2)	26.3 (5.0) **
**Disease severity,** $\bar{x}$ **(SD)**		
FACED score	2.03 (1.76)	1.92 (1.52)
EFACED score	2.85 (2.31)	2.45 (1.93) *
BSI	8.08 (4.79)	6.86 (4.22) **
Exacerbations	1.75 (1.98)	1.35 (1.44) **
Hospitalizations	0.76 (1.3)	0.51 (1.36) *
Charlson Index	1.92 (1.69)	1.75 (1.47)
Chronic colonization by PA, N (%)	30 (25)	171 (25)
**Smoking history**		
Never smokers, N (%)	151 (64)	368 (65)
Current smokers, N (%)	20 (8)	45 (8)
Ex-smokers, N (%)	65 (28)	154 (27)
Packs-year, $\bar{x}$ (SD)	10.1 (19.4)	7.8 (15.6)
**Lung function,** $\bar{x}$ **(SD)**		
FEV_1_, % predicted	74 (27)	78 (23), *p* = 0.07
FVC, % predicted	85 (24)	87 (21)
FEV_1_/FVC, %	68 (13)	71 (11) **
DLCO, % predicted	79 (22)	87 (23) *
KCO, % predicted	78 (33)	78 (39)
RV, % predicted	134 (38)	134 (49)
TLC, % predicted	98 (20)	101 (18)
RV/TLC, %	52 (10)	49 (12), *p* = 0.084

Continuous variables are presented as the mean (standard deviation), while categorical variables are presented as the number of patients in each group, along with the percentage for the study group. Definitions of the abbreviations: *N*, number; kg, kilograms; m, meters; BMI, body mass index; BSI, bronchiectasis severity index; PA, *Pseudomonas aeruginosa*; FEV_1_, forced expiratory volume in the first second; FVC, forced vital capacity; RV, residual volume; TLC, total lung capacity; DLco, carbon monoxide transfer; K_CO_, Krogh transfer factor. Statistical analyses and significance: *, *p* < 0.05; **, *p* < 0.01; ***, *p* < 0.001 between the patient groups.

**Table 2:** (**A**) Inflammatory and nutritional parameters in the two clusters of patients. (**B**) The inflammatory and nutritional parameters in the two clusters of bronchiectasis patients, excluding those with COPD.

**(A)**		
	**Below-Threshold Cluster**	**Above-Threshold Cluster**
	**(*N* = 270)**	**(*N* = 636)**
**Blood parameters,** $\bar{x}$ **(SD)**		
Total leukocytes, cells/µL	7.68 (3.29) × 10^3^	7.5 (2.53) × 10^3^
Total neutrophils, cells/µL	5.28 (3.09) × 10^3^	4.49 (2.21) × 10^3^ ***
Neutrophils, %	65.41 (13.1)	58.11 (10.36) ***
Total lymphocytes, cells/µL	1.72 (0.8) × 10^3^	2.12 (0.74) × 10^3^ ***
Lymphocytes, %	25.29 (12.11)	29.7 (9.61) ***
Total eosinophils, cells/µL	0.06 (0.05) × 10^3^	0.26 (0.17) × 10^3^ ***
Eosinophils, %	0.79 (0.65)	3.6 (2.23) ***
Platelets, cells/µL	246 (81) × 10^3^	256 (73) × 10^3^
IgE, U/mL	94.41 (422.47)	152.42 (404.19)
IgE aspergillus, KU/L	6.37 (17.65)	3.77 (12.33)
IgM, mg/dL	86.1 (103.56)	100.6 (101.17)
IgA, mg/dL	191.08 (153.45)	216.63 (147.07)
IgG, mg/dL	777.44 (545.66)	945.15 (516.38) **
Alpha-1 antitrypsin, mg/dL	132.48 (34.76)	133.77 (34.3)
CRP, mg/dL	2.81 (5.69)	2.8 (5.64)
ESR, mm/h	17.4 (16.42)	16.94 (15.4)
Fibrinogen, mg/dL	435 (139)	419 (134)
Hemoglobin, g/dL	13.36 (1.66)	13.77 (1.45) ***
Hematocrit, %	40.54 (4.78)	41.94 (4.09) ***
Creatinine, mg/dL	0.83 (0.53)	0.83 (0.42)
Total proteins, g/dL	6.9 (0.69)	7.04 (0.58) *
Albumin, g/dL	4.11 (0.46)	4.24 (0.41) **
**(B)**		
	**Below-Threshold Cluster**	**Above-Threshold Cluster**
	**(*N* = 236)**	**(*N* = 567)**
**Blood Parameters,** $\bar{x}$ **(SD)**		
Total leukocytes, cells/µL	7.44 (3.19) × 103	7.39 (2.4) × 103
Total neutrophils, cells/µL	5.04 (2.98) × 103	4.4 (2.09) × 103 **
Neutrophils, %	64.45 (12.94)	57.85 (10.31) ***
Total lymphocytes, cells/µL	1.73 (0.78) × 103	2.12 (0.74) × 103***
Lymphocytes, %	26.13 (11.99)	30.01 (9.55) ***
Total eosinophils, cells/µL	0.05 (0.03) × 103	0.26 (0.18) × 103 ***
Eosinophils, %	0.84 (0.65)	3.63 (2.26) ***
Platelets, cells/µL	247.68 (82.7) × 103	257.31 (72.26) × 103
IgE, U/mL	86.8 (442.43)	146.66 (400.49)
IgE aspergillus, KU/L	6.45 (17.99)	3.89 (12.64)
IgM, mg/dL	87.25 (108)	95.48 (81.91)
IgA, mg/dL	185.04 (147.07)	211.7 (146.35)
IgG, mg/dL	789.22 (546.8)	936.31 (511.64) **
Alpha-1 antitrypsin, mg/dL	132.84 (36.23)	132.75 (34.28)
CRP, mg/dL	2.8 (5.85)	2.65 (5.31)
ESR, mm/h	17.66 (17.12)	17.07 (15.18)
Fibrinogen, mg/dL	430 (140)	415 (133)
Hemoglobin, g/dL	13.3 (1.63)	13.69 (1.39) **
Hematocrit, %	40.37 (4.67)	41.72 (3.84) ***
Creatinine, mg/dL	0.83 (0.56)	0.82 (0.43)
Total proteins, g/dL	6.95 (0.67)	7.07 (0.55) *
Albumin, g/dL	4.16 (0.44)	4.26 (0.4) *

Continuous variables are presented as the mean (standard deviation) for the study group. Definitions of the abbreviations: IgE, immunoglobulin E; IgE aspergillus, IgE aspergillus fumigatus; IgM, immunoglobulin M; IgA, immunoglobulin A; IgG, immunoglobulin G; CRP, C-reactive protein; ESR, erythrocyte sedimentation rate; L, liter; mL, milliliter; g, grams; dL, deciliter; uL, microliter; mg, milligrams; mm, millimeters; h, hour; ng, nanogram. Statistical analyses and significance: *, *p* < 0.05; **, *p* < 0.01; ***, *p* < 0.001 between the patient groups.

**Table 3:** Etiology of the non-CF bronchiectasis patients.

Etiology, N, %	Total	Below-Threshold Cluster	Above-Threshold Cluster
Post-infectious	343, 37.9%	113, 42%	230, 36.2%
Tuberculosis	122, 13.5%	42, 15.6%	80, 12.6%
Childhood infections	83, 9.2%	26, 9.6%	57, 9%
Necrotizing pneumonia	29, 3.2%	6, 2.2%	23, 3.6%
Non-tuberculous mycobacteria	10, 1.1%	3, 1.1%	7, 1.1%
Fungal infections	4, 0.4%	2, 0.7%	2, 0.3%
Others	658, 72.6%	191, 70.7%	467, 73.4%
COPD	103, 11.4%	34, 12.6%	69, 10.8%
Asthma	90, 9.9%	24, 8.9%	66, 10.4%
Hyperimmune response	3, 0.3%	1, 0.4%	2, 0.3%
Vasculitis	2, 0.2%	0	2, 0.3%
Systemic disorders	78, 8.6%	17, 6.3%	61, 9.6%
Immunodeficiencies	42, 4.6%	13, 4.8%	29, 4.6%
Inflammatory pneumonitis	21, 2.3%	7, 2.6%	14, 2.2%
Congenital malformations	9, 1%	1, 0.4%	8, 1.3%
Inflammatory bowel diseases	9, 1%	2, 0.7%	7, 1.1%
Obliterative bronchiolitis	6, 0.7%	3, 1.1%	3, 0.5%
Unknown etiology	166, 18.3%	45, 16.7%	121, 19.0%
Other etiologies	34, 3.8%	10, 3.7%	24, 3.8%

Absolute number of patients and percentage for each etiologic condition. Definitions of the abbreviations: N, number; %, percentage; COPD: chronic obstructive pulmonary disease.

## Data Availability

The datasets generated and analyzed during the current study are available from the corresponding author on reasonable request.
